# Supplementary material for: Modeling glioblastoma heterogeneity as a dynamic network of cell states
Source: Mol Syst Biol. 2021 Sep 16;17(9):e10105. doi: 10.15252/msb.202010105 (PMC8444284; doi:10.15252/msb.202010105)
Supplement: Supplementary file 6 — Source Data for Figure 5 [file MSB-17-e10105-s004.zip › Figure5A_sourcedata/GSEA_3071/nosigdb_stateIII.GseaPreranked.1622795155562/RICHARDS_NOWAKOWSKI_SCIENCE_2017_U3_UPREG.html]

Details for gene set RICHARDS\_NOWAKOWSKI\_SCIENCE\_2017\_U3\_UPREG[GSEA]

|  || Dataset | state33071 |
| Phenotype | NoPhenotypeAvailable |
| Upregulated in class | na\_pos |
| GeneSet | RICHARDS\_NOWAKOWSKI\_SCIENCE\_2017\_U3\_UPREG |
| Enrichment Score (ES) | 0.20831157 |
| Normalized Enrichment Score (NES) | NaN |
| Nominal p-value | NaN |
| FDR q-value | 1.0 |
| FWER p-Value | 0.0 |
Table: GSEA Results Summary

  

Fig 1: Enrichment plot: RICHARDS\_NOWAKOWSKI\_SCIENCE\_2017\_U3\_UPREG      
 Profile of the Running ES Score & Positions of GeneSet Members on the Rank Ordered List

  

| PROBE | GENE SYMBOL | GENE\_TITLE | RANK IN GENE LIST | RANK METRIC SCORE | RUNNING ES | CORE ENRICHMENT || 1 | EIF5A |  |  | 1 | 0.691 | 0.0065 | Yes |
| 2 | RPL31 |  |  | 2 | 0.687 | 0.0133 | Yes |
| 3 | C1orf56 |  |  | 3 | 0.666 | 0.0199 | Yes |
| 4 | RPL38 |  |  | 5 | 0.638 | 0.0259 | Yes |
| 5 | RPS29 |  |  | 7 | 0.630 | 0.0319 | Yes |
| 6 | RPL27 |  |  | 11 | 0.587 | 0.0368 | Yes |
| 7 | NDUFAF2 |  |  | 15 | 0.567 | 0.0416 | Yes |
| 8 | RPL35 |  |  | 17 | 0.549 | 0.0468 | Yes |
| 9 | RPS25 |  |  | 19 | 0.527 | 0.0517 | Yes |
| 10 | RPL24 |  |  | 21 | 0.524 | 0.0566 | Yes |
| 11 | RPL22 |  |  | 22 | 0.520 | 0.0617 | Yes |
| 12 | RPL19 |  |  | 24 | 0.517 | 0.0666 | Yes |
| 13 | EEF1B2 |  |  | 28 | 0.501 | 0.0707 | Yes |
| 14 | RPL28 |  |  | 32 | 0.498 | 0.0748 | Yes |
| 15 | UBA52 |  |  | 33 | 0.494 | 0.0797 | Yes |
| 16 | RPS13 |  |  | 35 | 0.490 | 0.0842 | Yes |
| 17 | RPLP2 |  |  | 36 | 0.489 | 0.0890 | Yes |
| 18 | TXN |  |  | 47 | 0.465 | 0.0909 | Yes |
| 19 | RPS20 |  |  | 49 | 0.463 | 0.0952 | Yes |
| 20 | RPL5 |  |  | 51 | 0.461 | 0.0995 | Yes |
| 21 | NDUFA6 |  |  | 54 | 0.458 | 0.1035 | Yes |
| 22 | RPS5 |  |  | 57 | 0.454 | 0.1074 | Yes |
| 23 | RPL37A |  |  | 64 | 0.448 | 0.1102 | Yes |
| 24 | PSMA7 |  |  | 78 | 0.435 | 0.1109 | Yes |
| 25 | C6orf48 |  |  | 81 | 0.430 | 0.1146 | Yes |
| 26 | YWHAE |  |  | 85 | 0.427 | 0.1180 | Yes |
| 27 | NPM1 |  |  | 88 | 0.421 | 0.1216 | Yes |
| 28 | RPL10A |  |  | 94 | 0.414 | 0.1244 | Yes |
| 29 | RPL35A |  |  | 96 | 0.413 | 0.1282 | Yes |
| 30 | PFDN1 |  |  | 97 | 0.413 | 0.1322 | Yes |
| 31 | MRPL52 |  |  | 99 | 0.411 | 0.1360 | Yes |
| 32 | RPS27 |  |  | 132 | 0.388 | 0.1312 | Yes |
| 33 | COX6B1 |  |  | 134 | 0.387 | 0.1347 | Yes |
| 34 | COX7B |  |  | 135 | 0.386 | 0.1385 | Yes |
| 35 | GNG5 |  |  | 139 | 0.382 | 0.1415 | Yes |
| 36 | RPL21 |  |  | 141 | 0.382 | 0.1450 | Yes |
| 37 | NDUFA1 |  |  | 143 | 0.381 | 0.1485 | Yes |
| 38 | COX6A1 |  |  | 145 | 0.379 | 0.1519 | Yes |
| 39 | TMSB10 |  |  | 148 | 0.376 | 0.1551 | Yes |
| 40 | PPDPF |  |  | 157 | 0.369 | 0.1566 | Yes |
| 41 | SET |  |  | 163 | 0.366 | 0.1588 | Yes |
| 42 | NME1 |  |  | 166 | 0.364 | 0.1619 | Yes |
| 43 | NEDD8 |  |  | 168 | 0.362 | 0.1652 | Yes |
| 44 | UBL5 |  |  | 182 | 0.352 | 0.1651 | Yes |
| 45 | PSME1 |  |  | 190 | 0.347 | 0.1666 | Yes |
| 46 | DAP |  |  | 193 | 0.342 | 0.1695 | Yes |
| 47 | NUDC |  |  | 226 | 0.325 | 0.1640 | Yes |
| 48 | ST13 |  |  | 227 | 0.325 | 0.1672 | Yes |
| 49 | RPS2 |  |  | 228 | 0.325 | 0.1704 | Yes |
| 50 | SF3B6 |  |  | 235 | 0.319 | 0.1719 | Yes |
| 51 | BUD31 |  |  | 237 | 0.316 | 0.1748 | Yes |
| 52 | MRPL57 |  |  | 240 | 0.315 | 0.1773 | Yes |
| 53 | MZT2B |  |  | 244 | 0.313 | 0.1796 | Yes |
| 54 | RPL23 |  |  | 250 | 0.310 | 0.1813 | Yes |
| 55 | RPS16 |  |  | 256 | 0.308 | 0.1830 | Yes |
| 56 | NDUFA3 |  |  | 260 | 0.305 | 0.1852 | Yes |
| 57 | RPL41 |  |  | 265 | 0.303 | 0.1871 | Yes |
| 58 | RPL9 |  |  | 269 | 0.302 | 0.1893 | Yes |
| 59 | RPS4Y1 |  |  | 272 | 0.301 | 0.1917 | Yes |
| 60 | HSPE1 |  |  | 279 | 0.297 | 0.1930 | Yes |
| 61 | CDC42 |  |  | 289 | 0.295 | 0.1934 | Yes |
| 62 | TPM4 |  |  | 306 | 0.290 | 0.1919 | Yes |
| 63 | BANF1 |  |  | 317 | 0.287 | 0.1921 | Yes |
| 64 | SKA2 |  |  | 343 | 0.280 | 0.1880 | Yes |
| 65 | BTF3 |  |  | 344 | 0.279 | 0.1908 | Yes |
| 66 | RPS11 |  |  | 358 | 0.272 | 0.1899 | Yes |
| 67 | C11orf58 |  |  | 365 | 0.271 | 0.1910 | Yes |
| 68 | PDRG1 |  |  | 378 | 0.268 | 0.1904 | Yes |
| 69 | FKBP3 |  |  | 387 | 0.264 | 0.1908 | Yes |
| 70 | CHMP3 |  |  | 401 | 0.259 | 0.1898 | Yes |
| 71 | CTTN |  |  | 406 | 0.258 | 0.1913 | Yes |
| 72 | SEC22B |  |  | 410 | 0.256 | 0.1930 | Yes |
| 73 | ARPC4 |  |  | 416 | 0.255 | 0.1941 | Yes |
| 74 | RBX1 |  |  | 418 | 0.254 | 0.1964 | Yes |
| 75 | CWC15 |  |  | 422 | 0.253 | 0.1981 | Yes |
| 76 | HNRNPH1 |  |  | 426 | 0.252 | 0.1997 | Yes |
| 77 | NDUFB4 |  |  | 427 | 0.252 | 0.2022 | Yes |
| 78 | NDUFB8 |  |  | 428 | 0.251 | 0.2047 | Yes |
| 79 | HK2 |  |  | 432 | -0.250 | 0.2064 | Yes |
| 80 | SNHG12 |  |  | 435 | -0.250 | 0.2083 | Yes |
| 81 | SERGEF |  |  | 445 | -0.251 | 0.2083 | Yes |
| 82 | PPFIBP1 |  |  | 462 | -0.252 | 0.2065 | No |
| 83 | PLOD2 |  |  | 521 | -0.255 | 0.1932 | No |
| 84 | C5orf15 |  |  | 536 | -0.256 | 0.1919 | No |
| 85 | BCAP31 |  |  | 542 | -0.256 | 0.1931 | No |
| 86 | TLE1 |  |  | 555 | -0.257 | 0.1924 | No |
| 87 | POGLUT1 |  |  | 580 | -0.259 | 0.1884 | No |
| 88 | ETNK1 |  |  | 605 | -0.260 | 0.1845 | No |
| 89 | ARGLU1 |  |  | 633 | -0.261 | 0.1797 | No |
| 90 | TM9SF2 |  |  | 664 | -0.263 | 0.1741 | No |
| 91 | NR6A1 |  |  | 683 | -0.264 | 0.1719 | No |
| 92 | TRPS1 |  |  | 690 | -0.264 | 0.1728 | No |
| 93 | LINC01138 |  |  | 747 | -0.267 | 0.1602 | No |
| 94 | UNC50 |  |  | 763 | -0.267 | 0.1588 | No |
| 95 | ZFP30 |  |  | 776 | -0.268 | 0.1582 | No |
| 96 | DNAJC3 |  |  | 780 | -0.268 | 0.1600 | No |
| 97 | DEDD2 |  |  | 787 | -0.269 | 0.1610 | No |
| 98 | CHD1 |  |  | 812 | -0.270 | 0.1572 | No |
| 99 | EMC10 |  |  | 836 | -0.271 | 0.1536 | No |
| 100 | RAD52 |  |  | 838 | -0.271 | 0.1560 | No |
| 101 | ODF2L |  |  | 894 | -0.274 | 0.1438 | No |
| 102 | TAF1C |  |  | 903 | -0.274 | 0.1443 | No |
| 103 | ADCY3 |  |  | 932 | -0.276 | 0.1394 | No |
| 104 | HELLS |  |  | 970 | -0.278 | 0.1321 | No |
| 105 | EFNA5 |  |  | 1018 | -0.280 | 0.1221 | No |
| 106 | UBR4 |  |  | 1041 | -0.281 | 0.1189 | No |
| 107 | SULT1C4 |  |  | 1050 | -0.282 | 0.1195 | No |
| 108 | SLC38A2 |  |  | 1055 | -0.282 | 0.1212 | No |
| 109 | WWTR1 |  |  | 1080 | -0.284 | 0.1175 | No |
| 110 | GKAP1 |  |  | 1083 | -0.284 | 0.1198 | No |
| 111 | FOXO3 |  |  | 1088 | -0.284 | 0.1215 | No |
| 112 | TRIM59 |  |  | 1110 | -0.285 | 0.1186 | No |
| 113 | USP19 |  |  | 1145 | -0.287 | 0.1122 | No |
| 114 | MTRR |  |  | 1166 | -0.288 | 0.1096 | No |
| 115 | VPS13B |  |  | 1183 | -0.289 | 0.1081 | No |
| 116 | TMEM165 |  |  | 1201 | -0.290 | 0.1063 | No |
| 117 | FAM228B |  |  | 1209 | -0.291 | 0.1073 | No |
| 118 | RMI2 |  |  | 1249 | -0.292 | 0.0996 | No |
| 119 | FSTL1 |  |  | 1254 | -0.293 | 0.1014 | No |
| 120 | PLEKHH2 |  |  | 1282 | -0.294 | 0.0970 | No |
| 121 | RFX2 |  |  | 1293 | -0.295 | 0.0972 | No |
| 122 | AVIL |  |  | 1296 | -0.295 | 0.0995 | No |
| 123 | SLC4A7 |  |  | 1314 | -0.295 | 0.0978 | No |
| 124 | TUBG2 |  |  | 1327 | -0.296 | 0.0975 | No |
| 125 | RIMKLB |  |  | 1365 | -0.298 | 0.0904 | No |
| 126 | C9orf72 |  |  | 1382 | -0.299 | 0.0890 | No |
| 127 | LAPTM4A |  |  | 1391 | -0.299 | 0.0898 | No |
| 128 | CDON |  |  | 1423 | -0.301 | 0.0843 | No |
| 129 | MMS22L |  |  | 1494 | -0.306 | 0.0683 | No |
| 130 | CALR |  |  | 1498 | -0.306 | 0.0705 | No |
| 131 | ETV6 |  |  | 1500 | -0.306 | 0.0733 | No |
| 132 | WNT5A |  |  | 1531 | -0.308 | 0.0681 | No |
| 133 | MTRNR2L8 |  |  | 1536 | -0.308 | 0.0701 | No |
| 134 | INPP5F |  |  | 1558 | -0.310 | 0.0675 | No |
| 135 | TOB1 |  |  | 1573 | -0.310 | 0.0667 | No |
| 136 | KLF6 |  |  | 1596 | -0.312 | 0.0638 | No |
| 137 | GALT |  |  | 1597 | -0.312 | 0.0669 | No |
| 138 | ARHGAP21 |  |  | 1608 | -0.312 | 0.0672 | No |
| 139 | CECR2 |  |  | 1708 | -0.319 | 0.0435 | No |
| 140 | MSX1 |  |  | 1715 | -0.320 | 0.0450 | No |
| 141 | EAF1 |  |  | 1746 | -0.321 | 0.0400 | No |
| 142 | WDR76 |  |  | 1795 | -0.324 | 0.0302 | No |
| 143 | TEP1 |  |  | 1811 | -0.325 | 0.0293 | No |
| 144 | CCPG1 |  |  | 1832 | -0.326 | 0.0271 | No |
| 145 | SERINC1 |  |  | 1837 | -0.326 | 0.0293 | No |
| 146 | GLG1 |  |  | 1839 | -0.326 | 0.0322 | No |
| 147 | ZMYND8 |  |  | 1841 | -0.326 | 0.0351 | No |
| 148 | PDE5A |  |  | 1869 | -0.328 | 0.0310 | No |
| 149 | ADAM9 |  |  | 1870 | -0.328 | 0.0343 | No |
| 150 | ANKRD11 |  |  | 1878 | -0.328 | 0.0356 | No |
| 151 | TAF8 |  |  | 1892 | -0.329 | 0.0353 | No |
| 152 | NFKBIZ |  |  | 1903 | -0.330 | 0.0359 | No |
| 153 | HIP1 |  |  | 1905 | -0.330 | 0.0389 | No |
| 154 | SMC2 |  |  | 1963 | -0.333 | 0.0267 | No |
| 155 | KCNJ2 |  |  | 1974 | -0.334 | 0.0272 | No |
| 156 | ISYNA1 |  |  | 2010 | -0.336 | 0.0210 | No |
| 157 | SLC16A1 |  |  | 2012 | -0.336 | 0.0241 | No |
| 158 | UBAP1 |  |  | 2018 | -0.337 | 0.0261 | No |
| 159 | POLR3E |  |  | 2092 | -0.341 | 0.0096 | No |
| 160 | NINL |  |  | 2098 | -0.341 | 0.0116 | No |
| 161 | TMED10 |  |  | 2100 | -0.341 | 0.0147 | No |
| 162 | DCUN1D3 |  |  | 2108 | -0.342 | 0.0162 | No |
| 163 | MOAP1 |  |  | 2221 | -0.350 | -0.0108 | No |
| 164 | CCNDBP1 |  |  | 2319 | -0.359 | -0.0336 | No |
| 165 | FGD1 |  |  | 2326 | -0.360 | -0.0317 | No |
| 166 | INPP5B |  |  | 2327 | -0.360 | -0.0281 | No |
| 167 | HMGA2 |  |  | 2330 | -0.360 | -0.0251 | No |
| 168 | LIMCH1 |  |  | 2357 | -0.362 | -0.0286 | No |
| 169 | PFAS |  |  | 2386 | -0.364 | -0.0326 | No |
| 170 | HAS2 |  |  | 2400 | -0.365 | -0.0326 | No |
| 171 | EDEM1 |  |  | 2411 | -0.366 | -0.0317 | No |
| 172 | IRGQ |  |  | 2428 | -0.368 | -0.0324 | No |
| 173 | BCAT2 |  |  | 2448 | -0.369 | -0.0339 | No |
| 174 | GTF2IRD1 |  |  | 2501 | -0.373 | -0.0444 | No |
| 175 | IQGAP2 |  |  | 2538 | -0.376 | -0.0504 | No |
| 176 | NRDE2 |  |  | 2603 | -0.382 | -0.0641 | No |
| 177 | POLR1B |  |  | 2606 | -0.382 | -0.0608 | No |
| 178 | TMTC2 |  |  | 2616 | -0.383 | -0.0595 | No |
| 179 | LIN52 |  |  | 2622 | -0.384 | -0.0571 | No |
| 180 | SPTBN1 |  |  | 2647 | -0.385 | -0.0598 | No |
| 181 | STK35 |  |  | 2706 | -0.391 | -0.0717 | No |
| 182 | CLK2 |  |  | 2744 | -0.394 | -0.0778 | No |
| 183 | CLSTN3 |  |  | 2771 | -0.396 | -0.0810 | No |
| 184 | SFRP1 |  |  | 2839 | -0.402 | -0.0952 | No |
| 185 | ALCAM |  |  | 2853 | -0.403 | -0.0948 | No |
| 186 | SMC4 |  |  | 2872 | -0.405 | -0.0957 | No |
| 187 | GOLGA8B |  |  | 2885 | -0.407 | -0.0949 | No |
| 188 | SVIL |  |  | 2912 | -0.409 | -0.0979 | No |
| 189 | SERPINH1 |  |  | 2921 | -0.410 | -0.0961 | No |
| 190 | AASS |  |  | 2929 | -0.412 | -0.0939 | No |
| 191 | ZBTB21 |  |  | 2952 | -0.414 | -0.0958 | No |
| 192 | RTTN |  |  | 2999 | -0.420 | -0.1041 | No |
| 193 | CRLF3 |  |  | 3015 | -0.422 | -0.1040 | No |
| 194 | ZNF557 |  |  | 3040 | -0.425 | -0.1064 | No |
| 195 | PRR3 |  |  | 3048 | -0.425 | -0.1041 | No |
| 196 | POLI |  |  | 3052 | -0.426 | -0.1007 | No |
| 197 | GOLGA8A |  |  | 3053 | -0.426 | -0.0965 | No |
| 198 | CHPT1 |  |  | 3060 | -0.426 | -0.0939 | No |
| 199 | PTPRS |  |  | 3062 | -0.427 | -0.0900 | No |
| 200 | FANCD2 |  |  | 3065 | -0.427 | -0.0863 | No |
| 201 | HARS2 |  |  | 3087 | -0.430 | -0.0878 | No |
| 202 | SLC48A1 |  |  | 3094 | -0.430 | -0.0852 | No |
| 203 | ATRNL1 |  |  | 3109 | -0.432 | -0.0847 | No |
| 204 | ADAM19 |  |  | 3132 | -0.435 | -0.0864 | No |
| 205 | PRKD1 |  |  | 3146 | -0.437 | -0.0856 | No |
| 206 | ZBTB37 |  |  | 3153 | -0.438 | -0.0829 | No |
| 207 | KIAA1328 |  |  | 3158 | -0.439 | -0.0797 | No |
| 208 | NKIRAS1 |  |  | 3161 | -0.439 | -0.0759 | No |
| 209 | EEF2K |  |  | 3167 | -0.440 | -0.0729 | No |
| 210 | PAPOLG |  |  | 3201 | -0.442 | -0.0775 | No |
| 211 | SEMA5A |  |  | 3229 | -0.447 | -0.0804 | No |
| 212 | AREL1 |  |  | 3237 | -0.449 | -0.0779 | No |
| 213 | NR2C1 |  |  | 3268 | -0.454 | -0.0816 | No |
| 214 | ZNF136 |  |  | 3278 | -0.456 | -0.0795 | No |
| 215 | ARHGEF26 |  |  | 3304 | -0.461 | -0.0818 | No |
| 216 | ITM2B |  |  | 3314 | -0.463 | -0.0796 | No |
| 217 | TVP23B |  |  | 3323 | -0.466 | -0.0772 | No |
| 218 | MEST |  |  | 3363 | -0.473 | -0.0831 | No |
| 219 | CTH |  |  | 3386 | -0.478 | -0.0844 | No |
| 220 | TMEM67 |  |  | 3465 | -0.497 | -0.1007 | No |
| 221 | ZFHX4 |  |  | 3540 | -0.524 | -0.1156 | No |
| 222 | DCBLD2 |  |  | 3543 | -0.525 | -0.1110 | No |
| 223 | PLEKHA5 |  |  | 3554 | -0.528 | -0.1085 | No |
| 224 | SOX9 |  |  | 3560 | -0.531 | -0.1046 | No |
| 225 | THBS1 |  |  | 3599 | -0.547 | -0.1095 | No |
| 226 | VCL |  |  | 3603 | -0.551 | -0.1049 | No |
| 227 | TARBP1 |  |  | 3620 | -0.560 | -0.1037 | No |
| 228 | LSAMP |  |  | 3623 | -0.561 | -0.0987 | No |
| 229 | RFTN2 |  |  | 3654 | -0.582 | -0.1011 | No |
| 230 | COLEC12 |  |  | 3699 | -0.613 | -0.1070 | No |
| 231 | GNAS |  |  | 3714 | -0.630 | -0.1046 | No |
| 232 | RORA |  |  | 3716 | -0.630 | -0.0987 | No |
| 233 | DCN |  |  | 3723 | -0.633 | -0.0941 | No |
| 234 | STON2 |  |  | 3739 | -0.658 | -0.0917 | No |
| 235 | AKAP12 |  |  | 3740 | -0.660 | -0.0851 | No |
| 236 | FBLN1 |  |  | 3744 | -0.664 | -0.0794 | No |
| 237 | MMP16 |  |  | 3753 | -0.675 | -0.0749 | No |
| 238 | TGFBI |  |  | 3781 | -0.713 | -0.0752 | No |
| 239 | TFAP2B |  |  | 3792 | -0.729 | -0.0707 | No |
| 240 | MAP3K1 |  |  | 3796 | -0.737 | -0.0643 | No |
| 241 | PROM1 |  |  | 3854 | -0.846 | -0.0714 | No |
| 242 | LUM |  |  | 3861 | -0.870 | -0.0645 | No |
| 243 | COL6A1 |  |  | 3863 | -0.872 | -0.0561 | No |
| 244 | KCNQ1OT1 |  |  | 3866 | -0.885 | -0.0480 | No |
| 245 | SCRG1 |  |  | 3869 | -0.904 | -0.0396 | No |
| 246 | CTGF |  |  | 3901 | -1.185 | -0.0363 | No |
| 247 | COL1A1 |  |  | 3902 | -1.191 | -0.0246 | No |
| 248 | IGFBP2 |  |  | 3917 | -1.404 | -0.0145 | No |
| 249 | PDGFRA |  |  | 3928 | -1.773 | 0.0003 | No |
Table: GSEA details [plain text format]

  

Fig 2: RICHARDS\_NOWAKOWSKI\_SCIENCE\_2017\_U3\_UPREG: Random ES distribution      
 Gene set null distribution of ES for **RICHARDS\_NOWAKOWSKI\_SCIENCE\_2017\_U3\_UPREG**
